# Noninvasive Identification of Viable Cell Populations in Docetaxel-Treated Breast Tumors Using Ferritin-Based Magnetic Resonance Imaging

**DOI:** 10.1371/journal.pone.0052931

**Published:** 2013-01-02

**Authors:** YoonSeok Choi, Hoe Suk Kim, Kyoung-Won Cho, Kyung-Min Lee, Yoon Jung Yi, Sung-Jong Eun, Hyun Jin Kim, Jisu Woo, Seung Hong Choi, Taeg-Keun Whangbo, ChulSoo Choi, Dong-Young Noh, Woo Kyung Moon

**Affiliations:** 1 Department of Radiology, Seoul National University Hospital, Seoul, Korea; 2 Department of Biomedical Science, College of Medicine, Seoul National University, Seoul, Korea; 3 The Institute of Radiation Medicine, Medical Research Center, Seoul National University, Seoul, Korea; 4 Department of Surgery, Seoul National University Hospital, Seoul, Korea; 5 Institute of LeeGilYeo Cancer and Diabetes Center, Gachon University of Medical Science, Incheon, Korea; 6 Department of Computer Science, Gachon University, Seongnam, Korea; University of Chicago, United States of America

## Abstract

**Background:**

Cancer stem cells (CSCs) are highly tumorigenic and are responsible for tumor progression and chemoresistance. Noninvasive imaging methods for the visualization of CSC populations within tumors *in vivo* will have a considerable impact on the development of new CSC-targeting therapeutics.

**Methodology/Principal Findings:**

In this study, human breast cancer stem cells (BCSCs) transduced with dual reporter genes (human ferritin heavy chain [FTH] and enhanced green fluorescence protein [EGFP]) were transplanted into NOD/SCID mice to allow noninvasive tracking of BCSC-derived populations. No changes in the properties of the BCSCs were observed due to ferritin overexpression. Magnetic resonance imaging (MRI) revealed significantly different signal intensities (R_2_* values) between BCSCs and FTH-BCSCs *in vitro* and *in vivo*. In addition, distinct populations of pixels with high R_2_* values were detected in docetaxel-treated FTH-BCSC tumors compared with control tumors, even before the tumor sizes changed. Histological analysis revealed that areas showing high R_2_* values in docetaxel-treated FTH-BCSC tumors by MRI contained EGFP+/FTH+ viable cell populations with high percentages of CD44+/CD24− cells.

**Conclusions/Significance:**

These findings suggest that ferritin-based MRI, which provides high spatial resolution and tissue contrast, can be used as a reliable method to identify viable cell populations derived from BCSCs after chemotherapy and may serve as a new tool to monitor the efficacy of CSC-targeting therapies *in vivo*.

## Introduction

Since the first identification of breast cancer stem cells (BCSCs) from human tumor samples using CD44+/CD24− markers by Al-Hajj et al., the role of BCSCs in tumor progression and therapeutic resistance has been actively investigated to develop better anti-cancer treatment strategies [1], [2]. In breast cancer patients, administration of chemotherapy or radiation therapy increases the fraction of CD44+/CD24− tumor cells and augments mammosphere formation *in vitro* and tumorigenicity in xenotransplantation models [3], [4]. The presence of BCSC markers or gene expression signatures correlates with poor prognosis in clinical tumor samples [5], [6]. The therapeutic resistance of BCSCs is associated with alterations in self-renewal and cell fate signaling path ways, including Notch, Wnt, Hedgehog, and HER-2 [2]. New therapeutic regimens using single agents or a combination of various drugs that target BCSCs are now under preclinical or clinical trials. Monitoring the efficacy of cancer stem cell (CSC) therapeutics *in vivo*, however, is challenging because the conventional method of measuring tumor size is inadequate as an endpoint [7]. *In vivo* identification of BCSCs using cellular imaging techniques will be extremely useful for this purpose because the efficacy of treatment depends more on the fraction of viable cancer cells in the tumor [8], [9].

*In vivo* imaging methods, including intravital microscopy, fluorescent imaging, luciferase imaging, positron emission tomography (PET), and magnetic resonance imaging (MRI), have been used to track cancer cells and monitor treatment response [10]–[15]. However, there have only been a few reports of *in vivo* imaging of CSCs in different types of tumors [16], [17]. Snyder et al. analyzed CSCs using quantum dot-conjugated antibodies against CD44v6 and CD24 in tumors and suggested the possibility of applying this approach to BCSC imaging [12]. Vlashi et al. demonstrated reduced 26S proteasome activity in CSCs originating from glioma cells and monitored these CSCs *in vivo* using a fluorescent protein (ZsGreen) fused to ornithine decarboxylase, which is a target of the 26S proteasome [13]. Liu et al. longitudinally monitored CSCs derived from breast cancer patients in an orthotopic xenograft mouse model using ubiquitin promoter-driven luciferase and showed the role of BCSCs in metastasis with imaging techniques [14]. Recently, Yoshii et al. showed that in a mouse colon carcinoma model, Cu-64-ATSM, a PET imaging agent, localizes preferentially in tumor regions with a high density of CD133+ cells with CSC characteristics [15]. However, *in vivo* imaging of BCSCs using MRI or PET has not been reported to date. MRI can provide tomographic or volumetric imaging of internal organs at high anatomical resolutions and soft tissue contrast without using ionizing radiation, which is not possible with other imaging modalities. Clinically, MRI is routinely used to identify and localize tumors before surgery and to monitor the response to treatment in breast cancer [18].

There are two approaches to track and image cells of interest *in vivo* with MRI. The first method uses a contrast agent as a labeling or targeting agent. To date, superparamagnetic iron oxide (SPIO) nanoparticles, due to their high relaxivity, have been the most widely used contrast agents for tracking and imaging diverse cells [19], [20]. With surface modification of SPIO nanoparticles, cells of interest can be targeted by an antibody, peptide, or nucleotide conjugation [21]. The presence of SPIO nanoparticles in the magnetic field leads to low signal intensities in T_2_ or T_2_* sensitive images. However, this method does not enable the long-term imaging of the cells of interest because the contrast agents become diluted as the cells divide, and the SPIO nanoparticle signals can accumulate in sites within tumors, where the cells are not viable [9]. The use of the MRI reporter gene ferritin can overcome these limitations [22]–[26]. The overexpression of ferritin enables cells to uptake more iron, and this reporter produces low signal intensities in MRI. As MRI reporters are stably expressed, even during cell division, they can be used for studying dynamic processes, *e.g*., the migration and invasion of cells of interest over an extended period of time and can also be useful for providing temporal and spatial information for anti-cancer treatment effects on a specific cell population. The number of cancer cells or level of tumor burden in deep tissues can be quantified by calculating R_2_* ( = 1/T_2_*) values from T_2_* mapping of MRI images [27], [28]. In addition, the introduction of optical reporter genes, such as EGFP or luciferase, and ferritin together allows for the analysis of cancer cells isolated from tumors in molecular biology and histology experiments.

In the present study, BCSCs isolated from human breast cancer specimens were transduced with MRI (human ferritin heavy chain, FTH) and fluorescence (enhanced green fluorescence protein, EGFP) dual reporter genes and transplanted into nonobese diabetic/severe combined immunodeficient (NOD/SCID) mice to noninvasively track BCSC-derived populations during tumor growth and monitor tumor responses after chemotherapy. An MRI evaluation of ferritin-overexpressing BCSCs (FTH-BCSCs) was performed, and the tumor response to chemotherapy was determined by quantification of the R_2_* values for entire tumors. In addition, viable cells were identified and localized by volumetric MRI, and BCSC characteristics were investigated by histological analysis.

## Methods

### Ethics Statement

All of the procedures were performed following approval by the Institutional Review Board (IRB) at Seoul National University Hospital. IRB approval number is H-0502-142-007. The individual in this manuscript has given written informed consent (as outlined in PLOS consent form) with the Declaration of Helsinki to publish these case details.

In animal study, 6-week-old NOD/SCID female mice were used. The animal study was reviewed and approved by the Institutional Animal Care and Use Committee (IACUC; No.11-0105) of the Seoul National University Hospital, and the procedures for all animal experiments were performed according to IACUC guidelines.

### Isolation of Primary BCSCs and Establishment of FTH-BCSCs

The human breast cancer tissues were obtained in operating room within 30 minutes after excision from routine surgical procedures for breast cancer patients. After excision from the surgical procedures, tissues (ER-, PR-, HER2-) were minced into 1 mm^3^ sized pieces and digested with collagenases at 37°C for four hour. After rinsing with the proper amount of medium used for mammosphere culture, CD44+/CD24− cells were obtained with fluorescence-activated cell sorter (FACS-Aria, BD Biosciences) and maintained using an anchorage-independent culture method [29]. The BCSCs were incubated with DMEM mixed 3∶1 with Ham’s F12 medium (Invitrogen) supplemented with basic fibroblast growth factor (10 ng/ml; Millipore), epidermal growth factor (20 ng/ml; Invitrogen), leukemia inhibitory factor (10 ng/ml; Millipore), B27 supplement (Invitrogen) and antibiotic-antimycotic solution (Invitrogen) for mammosphere culture.

To establish the FTH-BCSCs, a lentivirus expressing myc-tagged FTH (myc-FTH) and EGFP driven by the cytomegalovirus (CMV) and phosphoglycerate kinase (PGK) promoters, respectively, was introduced into cells by incubating the cells with 10^6^–10^7^ transduction units/ml for 6–10 hours in the presence of 8 µg/ml polybrene. After three days of transduction, cells expressing EGFP were sorted using a FACS-Aria, expanded and used in all experiments.

### Analysis of Surface Markers on FTH-BCSCs

To analyze cell surface markers, 5×10^5^ BCSCs and FTH-BCSCs were dissociated from mammospheres and washed two times with phosphate buffered saline (PBS) containing 1% BSA. The cells were then incubated with anti-huCD44-phytoerythrin (PE), anti-huCD24-PE, anti-huCD90-APC, anti-huCD49f-PE, or anti-huCD34-PE antibodies (BD Biosciences) for one hour at 37°C. Cell-associated fluorescence was measured using a FACS-Calibur flow cytometer (BD Biosciences). The data were analyzed using CellQuest software (BD Biosciences).

### Western Blot Analysis

Cells were lysed in RIPA buffer with 1 mM phenylmethylsulfonyl fluoride and a protease inhibitor cocktail (Sigma-Aldrich Chemical Co.). The protein lysates were resolved by SDS-polyacrylamide gel electrophoresis (SDS-PAGE) for four hours at room temperature and were transferred to nitrocellulose membranes for two hours at 4°C. After blocking, the membranes were incubated with anti-FTH (Santa Cruz Biotechnology) and anti-EGFP (Santa Cruz Biotechnology) antibodies overnight at 4°C followed by incubation with HRP-conjugated antibodies (Santa Cruz Biotechnology) at room temperature for 30 minutes. Blots were visualized using enhanced chemiluminescence reagents (Amersham Biosciences).

### Immunocytochemistry of Mammospheres

Mammospheres were fixed in 4% paraformaldehyde in PBS. For the detection of myc-FTH expression, fixed mammospheres were incubated with anti-c-myc antibody (Santa Cruz Biotechnology) overnight at 4°C followed by incubation with the Alexa Fluor 594-conjugated anti-mouse IgG antibody (Invitrogen) at room temperature for one hour. Hoechst 33342 (Invitrogen) was used to visualize cell nuclei. Images were scanned and analyzed with a confocal laser microscope (LSM 5 META, Carl Zeiss).

### Measurement of Iron Loading

BCSCs or FTH-BCSCs (2×10^4^) were placed in 12-well Petri dishes, and 0 to 50 µM ferric ammonium citrate (FAC; Sigma-Aldrich Chemical Co.) was added to the culture medium for 4 days. Harvested cells were counted and subsequently lysed with 6 N HCl to extract total iron. The amount of total iron was determined using a total iron reagent kit (Pointe Scientific), and the average iron amount in a cell was calculated by dividing the total mean iron by the cell number.

### Mammosphere Assay

To compare the mammosphere-forming abilities of the BCSCs and FTH-BCSCs, dissociated cells from BCSC and FTH-BCSC mammospheres were seeded in 96-well plates at a density of 100 cells/well and incubated for four days. The average number of mammospheres was calculated by counting the number of mammospheres in a well.

### Cell Growth and Viability with Iron Supplementation

To evaluate the growth and viability of the cells under different iron supply conditions, the trypan blue exclusion assay and flow cytometric analysis with 7-amino-actinomycin D (7-AAD) (BD Pharmingen) were performed. For the cell growth assays, 3×10^4^ BCSCs or FTH-BCSCs were initially seeded in 12-well plates supplemented with FAC (up to 50 µM), and the average number of cells was calculated on days 2, 4, 6 and 8. For the cell viability assays, BCSCs and FTH-BCSCs were grown in medium supplemented with increasing amounts of FAC for five days. Next, both cell types were collected, incubated with 7-AAD for 5–10 minutes at 37°C and analyzed by flow cytometry.

### Tumor Formation and Tumor Volume Measurements

To compare the tumor formation abilities of BCSCs and FTH-BCSCs, 2×10^2^–1×10^6^ viable BCSCs and FTH-BCSCs were implanted into the mammary fat pads of NOD/SCID mice (BCSC tumors [n = 21]; FTH-BCSC tumors [n = 21]). Tumor formation was monitored up to eight weeks after implantation, depending on the number of injected cells.

To determine the volumes of the BCSC and FTH-BCSC xenograft tumors, a modified ellipsoidal formula for volume (volume = 1/2[length × width^2^]) was used, in which length was the measurement of the greatest longitudinal diameter and width was the greatest transverse diameter.

### Fluorescence Imaging

At three weeks post-injection, the fluorescence imaging of BCSC and FTH-BCSC tumors from a living mouse was assessed using a Maestro imaging system (CRi Inc.) with excitation and emission set at 445–490 nm and 515 nm, respectively. The filter was adjusted while the camera captured images, and the signals from the tumors were merged with a GFP-filtered image.

*Ex vivo* fluorescence imaging of BCSC and FTH-BCSC tumors excised from mice was performed by GFP fluorescence analysis (excitation: 470 nm, emission: 535 nm) using a Kodak Image Station 4000MM (Carestream Molecular Imaging).

### Docetaxel Treatment and Cytotoxicity Assays

To investigate the *in vitro* cytotoxicity of docetaxel on BCSCs and FTH-BCSCs, JC-1 staining and 3-2,5-diphenyltetrazolium bromide (MTT) assays were performed following treatment with docetaxel (1–10 nM, Sigma-Aldrich Chemical Co.) for 24 hours. To evaluate the changes in the mitochondrial membrane potential of both cell populations treated with docetaxel, the mitochondrial vital dye JC-1 (10 µg/ml, Invitrogen) was used. JC-1 aggregates with intense red fluorescence are known to accumulate in the intact mitochondria of healthy cells. When the mitochondrial membrane potential collapses in non-viable cells, JC-1 monomers fluoresce green. The percentage of cells with intact mitochondrial membranes was calculated by dividing the number of red fluorescence-positive cells by the total cell number.

### *In vivo* Docetaxel Treatment

The mice with BCSC (n = 18) and FTH-BCSC (n = 18) tumors were divided into docetaxel-treated (n = 19) and untreated control groups (n = 17). Docetaxel was treated when the tumor volumes had 70 mm^3^, usually 16 to 18 days after tumor cell implantation. In the docetaxel-treated groups, docetaxel (15 mg/kg) was injected into the tail vein of mice three times at intervals of 72 hours to evaluate the therapeutic effect of docetaxel on BCSC and FTH-BCSC tumors. The BCSC and FTH-BCSC tumors in the docetaxel-treated group are represented as BCSC Doc tumors (n = 10) and FTH-BCSC Doc tumors (n = 9), respectively. In the control group (BCSC tumors [n = 8]; FTH-BCSC tumors [n = 9]), saline was intravenously injected three times at the same intervals as those used in the docetaxel-treated groups.

### MRI Examination

All MRI studies were performed on a 9.4-T BrukerBiospec scanner (BrukerBiospin). A transmit-only volume coil and a four-channel surface coil (BrukerBiospin) were used for excitation and signal reception, respectively. For *in vitro* MRI, phantoms containing 2×10^6^ BCSCs and FTH-BCSCs, which were treated with or without 25 µM FAC for 4 days, were prepared. A multi-slice, multi-echo gradient echo sequence was used for *in vitro* T_2_* mapping. The parameters were as follows: matrix size = 256×256, repetition time (TR) = 5000 ms, slice thickness = 1 mm (no gap), flip angle = 90°, field of view (FOV) = 25×25 mm^2^, TE = 3.1–43.1 ms with a step size of 10 ms (five-point T_2_* mapping), 13 slices and 4 signal averages.

For *in vivo* MRI of mice bearing xenograft tumors, 1×10^6^ BCSCs or FTH-BCSCs were injected into the mammary fat pads of NOD/SCID mice. For whole-animal MRI, mice were anesthetized with isofluorane (1% in 100% oxygen). To stabilize the body temperature of the mice during MRI experiments, an animal warming system (BrukerBiospin) was used. Pre-treatment images were obtained three weeks after injection, and longitudinal follow-up images were obtained at 5 and 14 days after docetaxel treatment. *In vivo* data were acquired for fat saturation. The parameters were as follows: matrix size = 256×256, TR = 5000 ms, slice thickness = 1 mm (no gap), flip angle = 90°, FOV = 37×37 mm^2^, TE = 3.1–34.4 ms with a step size of 4.4 ms (eight-point T_2_* mapping), 17 slices and 1 signal average.

### MRI Data Analysis

All data post-processing was performed with Matlab (Mathworks Inc.). For both phantom and *in vivo* data, regions of interest (ROIs) were defined in each individual, imaging slices were acquired at the shortest TE and T_2_* maps of the ROIs were later estimated by pixel-by-pixel analyses across the multi-point MRI images, assuming single exponential decay. After performing the R_2_* analyses, R_2_* color maps were merged with the anatomical images acquired at the shortest TE. For *in vivo* data, volumetric tumor images were reconstructed from the entire set of 2D slices for each animal.

To investigate the differences in the R_2_* distribution among the animal groups, we obtained R_2_* histograms for each animal by including all pixels in the ROIs across all slices. Comparisons of the mean R_2_* values among the animal groups were performed based on the R_2_* distributions. Additionally, to represent the differences in the R_2_* distribution better, we defined the mean plus 3 standard deviation (mean +3 SDs) as a threshold (Protocol S1). The distribution of skewedness was analyzed using the threshold values of each group and subsequent pixel percentages. In addition, the mean R_2_* values of the pixels over the threshold values of each group of tumors were calculated for the comparison of the R_2_* value distribution of each group of tumors.

### Histochemical Analysis of Tumors

After MRI examination, the excised tissues were fixed with 10% buffered formalin and embedded in paraffin blocks. Tissues were sectioned into 4-µm-thick sections. Hematoxylin and eosin staining (H&E) was performed to distinguish the viable and nonviable cell populations within the tumors. Immunofluorescence staining for the surface markers CD44 and CD24 and the proliferative marker phospho-histone 3 (PH3) were performed. After incubation of tissue sections with a blocking solution for one hour at room temperature, primary antibodies against CD44 (Thermo Scientific), CD24 (Novus Biologicals), or PH3 (Novus Biologicals) were incubated overnight at 4°C. Next, fluorescence-conjugated antibodies, namely, anti-mouse IgG Alexa 488, 647 or anti-rabbit IgG Alexa 594 (Invitrogen), were incubated for 45 minutes at room temperature. Hoechst 33342 (Invitrogen) was used to visualize the cell nuclei. Immunofluorescence images of the tissue sections were obtained under a confocal laser microscope (Carl Zeiss) and a fluorescence microscope (Leica).

### Statistical Analysis

All data are presented as the mean ± SD for at least three independent experiments. The mean values of the data were statistically evaluated using ANOVA followed by an unpaired t-test. A Fisher’s exact test was used to analyze the differences in R_2_* value distributions. For all tests, *P*-values less than 0.05 or 0.01 were considered to be statistically significant.

## Results

### The Transduction of FTH and EGFP does not Alter BCSC Characteristics

**Figure 1:**
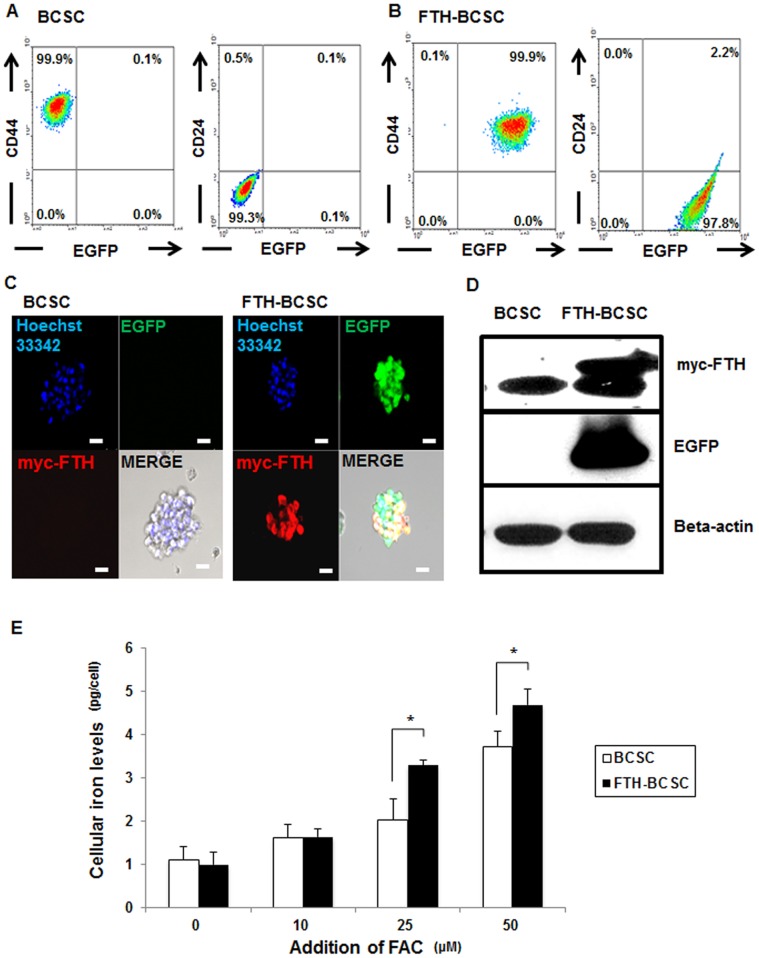
Establishment of breast cancer stem cells (FTH-BCSCs) that overexpress the myc-tagged heavy chain subunit of ferritin (myc-FTH) and EGFP. (A) Flow cytometric analysis of the surface markers, CD44 and CD24 in BCSCs isolated from human breast cancer specimens. (B) Flow cytometric analysis of the surface markers, CD44 and CD24 in FTH-BCSCs expressing myc-FTH and EGFP. (C) Immunocytochemistry of BCSCs and FTH-BCSCs (bar, 50 µm). (D) Western blot analysis for myc-FTH and EGFP in BCSCs and FTH-BCSCs. (E) Measurement of cellular iron in BCSCs and FTH-BCSCs treated with increasing concentrations of ferric ammonium citrate (FAC). The results were obtained from 4 independent experiments and are presented as means ± SD. *P<0.05.

BCSCs from a breast cancer patient with a CD44+/CD24− phenotype were isolated and maintained using mammosphere culture media (Figure 1A). To establish ferritin-overexpressing BCSCs, genes for myc-FTH and EGFP were transduced into cells with a lentiviral vector, and fluorescence-activated cell sorting (FACS) was used to collect the cells that expressed both myc-FTH and EGFP. The levels of BCSC markers (CD44+/CD24−) did not change in the FTH-BCSCs (Figure 1B). Immunofluorescence staining and western blot analysis revealed the expression of myc-FTH and EGFP in FTH-BCSCs (Figure 1C and D). Iron-loading abilities were investigated with the addition of up to 50 µM of FAC to both the FTH-BCSCs and BCSCs. The cellular iron levels of both groups increased in a dose-dependent manner, but the FTH-BCSCs had significantly higher iron levels compared with the BCSCs (Figure 1E; *P*<0.05 in 25 and 50 µM of FAC).

Next, we sought to determine whether the FTH-BCSCs retained their BCSC properties compared to control BCSCs. The mammosphere-forming abilities and surface marker expression of the BCSCs and FTH-BCSCs were compared. Four days after single-cell dissociation, BCSCs and FTH-BCSCs generated mammospheres, and no substantial differences were observed in the average numbers of mammospheres (Figure S1A). The human mammary stem cell marker CD49f was highly expressed in both BCSCs and FTH-BCSCs [30]. High levels of CD90 (mesenchymal lineage marker) and low levels of CD34 (hematopoietic stem cell marker) were observed, and the levels of all of these surface markers did not differ between the FTH-BCSCs and BCSCs (Figure S1B and C).

Next, we performed a tumor forming ability assay with serially diluted numbers of BCSCs and FTH-BCSCs to investigate whether ferritin overexpression affects tumorigenesis *in vivo* (Figure S2A). Various numbers of BCSCs or FTH-BCSCs were xenografted into the mammary fat pads of mice, and the incidence of the BCSC and FTH-BCSC tumors following engraftment with 2×10^2^ cells was approximately 25% with transplantation of more than 1×10^3^ BCSCs or FTH-BCSCs, resulting in 100% tumor formation (Table 1). The sizes of the BCSC and FTH-BCSC tumors were similar. *Ex vivo* fluorescence imaging showed the stable expression of EGFP in the FTH-BCSC tumors (Figure S2B). These results demonstrated that the FTH-BCSCs retained the characteristics of BCSCs, despite the overexpression of FTH and EGFP.

**Table 1:** Evaluation of the incidence of tumors in NOD/SCID mice implanted with different numbers of BCSCs and FTH-BCSCs.

Cell type	Cell numbers	Tumor incidence
**BCSC**	1×10^6^	3/3
	1×10^5^	6/6
	1×10^4^	4/4
	1×10^3^	4/4
	2×10^2^	1/4
**FTH-BCSC**	1×10^6^	3/3
	1×10^5^	6/6
	1×10^4^	4/4
	1×10^3^	4/4
	2×10^2^	1/4

We also investigated the effects of iron overload on the growth and viability of the BCSCs and FTH-BCSCs. The growth rates of the BCSCs and FTH-BCSCs were not affected by treatment with FAC (Figure S3A). Cytotoxicity was not observed in either group following treatment with 25 µM FAC (Figure S3B), which is regarded as the physiological iron concentration in mouse serum (250–350 µg/dl) [31]. Taken together, these findings demonstrate that the FTH-BCSCs retained the biological properties of the parent BCSCs and possessed an enhanced ability for iron storage, suggesting the feasibility of FTH-BCSC MRI *in vitro* and *in vivo*.

### FTH-BCSCs Exhibit a Significant Increase in R_2_* Values in MRI Compared to BCSCs

**Figure 2:**
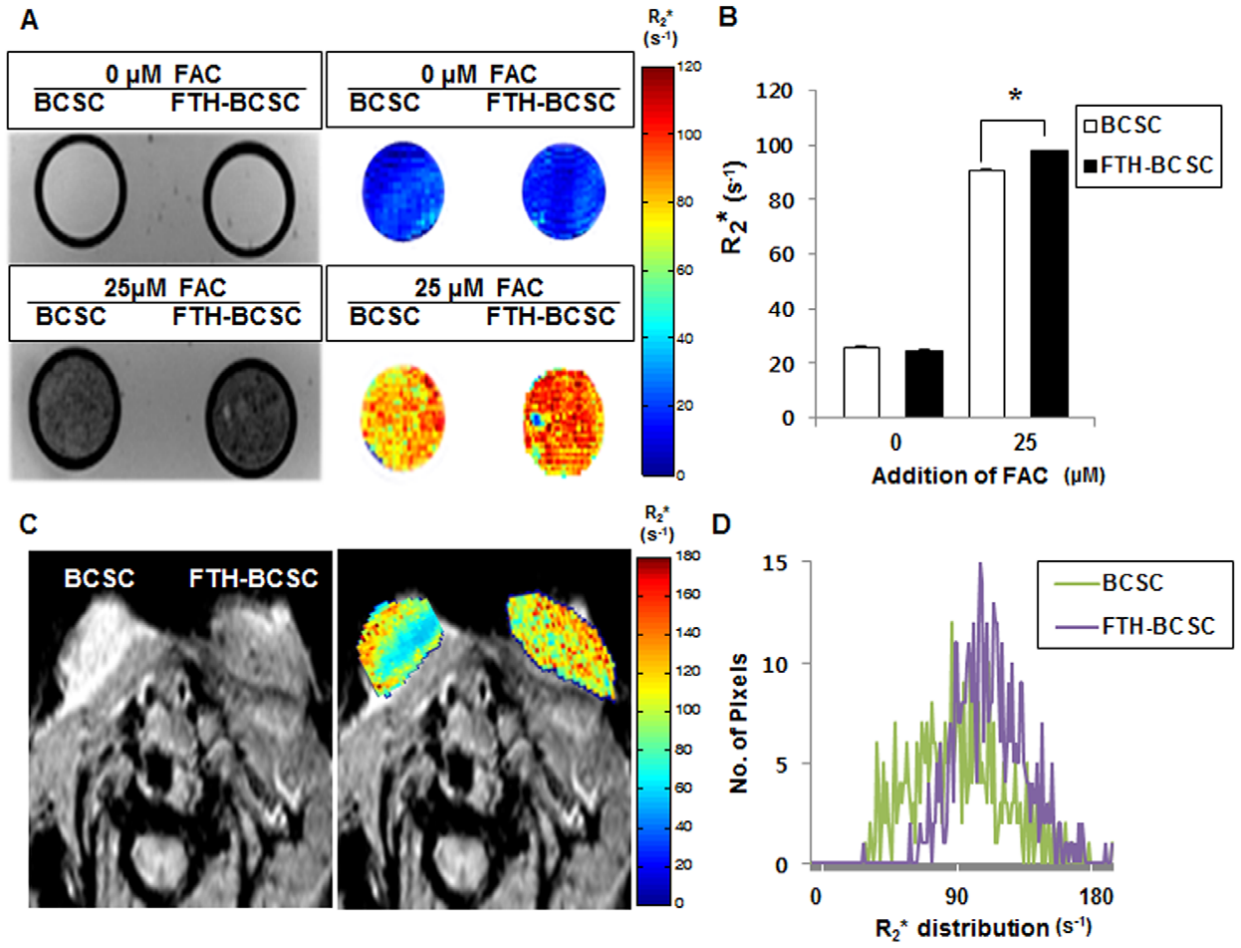
MRI images of phantom cells and xenograft tumors. (A) *In vitro* MRI images (left) and color-coded maps (right) of agarose phantoms of BCSCs and FTH-BCSCs treated with or without 25 µM FAC. (B) R_2_* values measured from MRI images of BCSC and FTH-BCSC phantoms. *P<0.05. (C) *In vivo* MRI images (left) and color-coded maps (right) of BCSC and FTH-BCSC xenograft tumors in the mammary fat pads of NOD/SCID mice. (D) The distributions of the R_2_* values obtained for the BCSC and FTH-BCSC tumors at 3 weeks post-transplantation.

To analyze the R_2_ * values of the BCSCs and FTH-BCSCs, cells were incubated with or without 25 µM FAC, and MRI images of cell phantoms were obtained (Figure 2A). The mean R_2_* values of the FTH-BCSCs (97.85±0.51 s^−1^) treated with 25 µM FAC were significantly higher than those of the BCSCs (90.72±0.21 s^−1^). However, the mean R_2_* values of the FTH-BCSCs and BCSCs in the absence of FAC were not different (Figure 2B).

Next, we investigated the effect of ferritin overexpression in a xenograft tumor model. Lower signal intensities were observed in the MRI images of FTH-BCSC tumors compared with BCSC tumors due to ferritin overexpression (Figure 2C, left). A color-coded map of BCSC and FTH-BCSC tumors revealed variable R_2_* values (Figure 2C, right). Although both the BCSC and FTH-BCSC tumors exhibited differences in the R_2_* values within the tumors, the FTH-BCSC tumors exhibited higher mean R_2_* values compared with the BCSC tumors, and the FTH-BCSC tumors exhibited shifted R_2_* distributions toward higher R_2_* values (Figure 2D; mean R_2_* values of BCSC tumors vs. FTH-BCSC tumors; 87.2±2.7 s^−1^ vs. 105.4±3.8 s^−1^; *P*<0.01).

### MRI Reveals Distinct Populations of Pixels with High R_2_* Values within Docetaxel-treated FTH-BCSC Tumors

Next, we evaluated the potential use of FTH-based MRI for monitoring the efficacy of an anti-cancer drug in xenografted tumors. We found that the growth of docetaxel-treated BCSC Doc and FTH-BCSC Doc tumors was attenuated compared to BCSC and FTH-BCSC tumors. Additionally, overexpression of FTH did not affect tumor growth (Figure S4).

**Figure 3:**
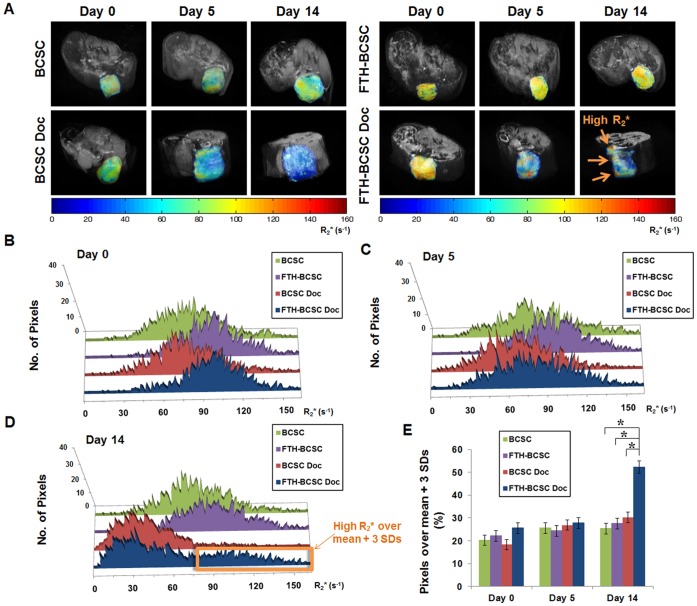
Volumetric MRI images and distribution of R_2_* values in control (BCSC and FTH-BCSC) and docetaxel-treated (BCSC Doc and FTH-BCSC Doc) xenograft tumors. (A) Follow-up volumetric MRI images of BCSC, BCSC Doc, FTH-BCSC and FTH-BCSC Doc tumors. (B-D) Distribution of R_2_* values in BCSC, FTH-BCSC, BCSC Doc and FTH-BCSC Doc tumors at day 0, 5 and 14. The orange box indicates the pixels over the threshold (mean +3 SDs) in the FTH-BCSC Doc tumors. E, analysis of the percentage of pixels over the threshold (mean +3SDs) in each tumor group. *P<0.05.

We subsequently performed slice-by-slice MRI analysis of tumors from each group and observed different distributions of tumor R_2_* values in volumetric images with longitudinal follow-up scans (Figure 3A and Figure S5). Before docetaxel treatment, the R_2_* value distribution between BCSC and BCSC Doc tumors and that between FTH-BCSC and FTH-BCSC Doc tumors were similar (Figure 3B and Table 2). At day 5 of docetaxel treatment, the mean R_2_* values of the BCSC Doc and FTH-BCSC Doc tumors were significantly decreased compared with the BCSC and FTH-BCSC tumors (*P*<0.05; Figure 3C and Table 2). At day 14 of docetaxel treatment, the BCSC Doc and FTH-BCSC Doc tumors exhibited lower mean R_2_* values compared with the BCSC and FTH-BCSC tumors (*P*<0.01; Figure 3D and Table 2). Notably, histogram analysis revealed that only the FTH-BCSC Doc tumors at day 14 of docetaxel treatment had distinct populations of pixels with high R_2_* values and a distribution of R_2_* values that was skewed compared with the other groups (Figure 3D). In the FTH-BCSC Doc tumors, pixels that represented a distinct population and were measured over the threshold value (mean +3SDs; Table S1) occupied approximately 50% of the total pixels, while the percentages of all the other groups were less than 30% (*P*<0.05, Figure 3E).

**Table 2:** Mean R_2_* values of control (BCSC and FTH-BCSC) and docetaxel-treated (BCSC Doc and FTH-BCSC Doc) xenograft tumors.

	Mean R_2_* value ±SD (sec^−1^)		*P* value
	BCSC	BCSC Doc	
Day 0	83.30±6.30	79.47±8.30	0.12
Day 5	**81.78±6.58**	**65.11±5.49**	0.03*
Day 14	**79.47±8.30**	**43.32±7.89**	0.001**
	**FTH-BCSC**	**FTH-BCSC Doc**	
Day 0	96.94±8.45	95.96±8.69	0.18
Day 5	**94.99±8.20**	**76.92±8.34**	0.02*
Day 14	**91.24±7.71**	**55.26±9.16**	0.001**

NOTE: Values in bold are statistically significant (*, *P*<0.05 and **, *P*<0.01).

### Histological Analysis Reveals that the Cell Populations with High R_2_* Values are Localized in Viable Areas of the FTH-BCSC Tumors

**Figure 4:**
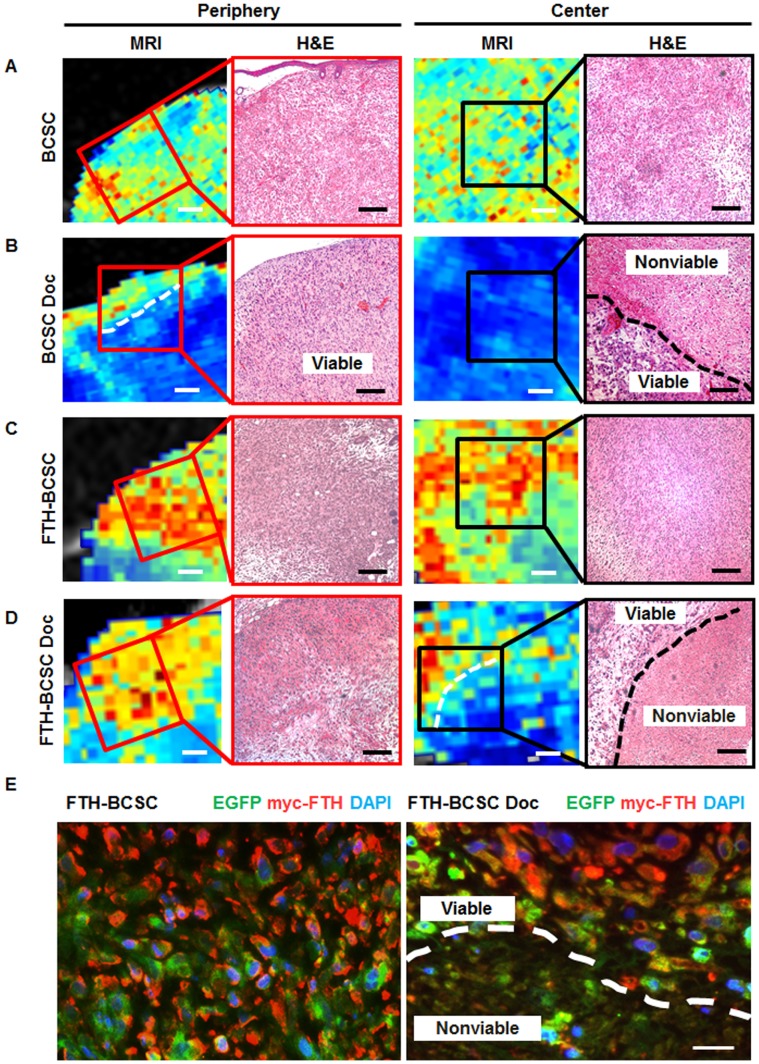
Histological analysis of the peripheral and central portions of control (BCSC and FTH-BCSC) and docetaxel-treated (BCSC Doc and FTH-BCSC Doc) xenograft tumors. (A–D) MRI images and H&E staining of BCSC, BCSC Doc, FTH-BCSC and FTH-BCSC Doc tumors obtained 4 weeks after the implantation. The red and the black boxes on the MRI images indicate the corresponding areas to H&E staining images of the periphery and the center of each tumor. The white dotted lines on the MRI images indicate the boundary where the R_2_* values were differed in the BCSC Doc and FTH-BCSC DOC tumors. The black dotted lines on H&E staining indicate the demarcation between viable and nonviable cells area in the central portions of the BCSC Doc and FTH-BCSC Doc tumors (x100; bar in H&E, 100 µm, bar in MRI, 500 µm). MRI images before magnification and cropping are in the Supplement (Figure S5). (E) Immunostaining of EGFP and myc-FTH in FTH-BCSC and FTH-BCSC Doc tumors. The white dotted line indicates the demarcation between the areas of viable and nonviable cells in the central portion of FTH-BCSC Doc tumors (x400; bar, 50 µm).

Next, we performed histological analysis to investigate whether ferritin-based MRI images reflects the tissue state after docetaxel treatment. MRI showed that the R_2_* value pixels were similarly distributed between the periphery and center of both BCSC and FTH-BCSC tumors and H&E staining revealed that most of the cells in the periphery and center of the BCSC and FTH-BCSC tumors were viable (Figure 4A and C). In contrast, MRI showed that the R_2_* value pixels were differently distributed between the periphery and center of BCSC Doc and FTH-BCSC Doc tumors and H&E staining revealed that the center of BCSC Doc tumors with low R_2_* values had both viable and nonviable cell populations (Figure 4B, right), whereas the center of the FTH-BCSC Doc tumors with mixed high and low R_2_* values matched those of the viable and nonviable cells within the tumors, respectively (Figure 4D, right). In addition, the periphery of BCSC Doc and FTH-BCSC Doc tumors with high R_2_* values had viable cells whereas viable cells were also found in the periphery of the BCSC Doc tumors with low R_2_* values (Figure 4B, left). The presence of viable cells in the FTH-BCSC and FTH-BCSC Doc tumors was confirmed with EGFP fluorescence (Figure S6). Additionally, we investigated the co-expression of myc-FTH and EGFP in the FTH-BCSC and FTH-BCSC Doc tumors and confirmed that the EGFP-positive cells had the myc-FTH expression (Figure 4E).

To clarify whether ferritin overexpression alters the therapeutic response to docetaxel, cell viability was evaluated by mitochondrial membrane potential analysis and MTT assay after treatment with docetaxel. Docetaxel induced a depolarization of the mitochondrial membrane potentials and reduced the intact mitochondria in both BCSCs and FTH-BCSCs (Figure S7A). A decrease in cell viability in the FTH-BCSCs and BCSCs were observed in a docetaxel dose-dependent manner using MTT assay (Figure S7B). There was no significant difference in the change of mitochondrial membrane potential and cell viability between FTH-BCSCs and BCSCs after treatment with docetaxel. Thus, we conclude that the ferritin overexpression did not affect the BCSCs’ response to docetaxel treatment.

### The Viable Cell Populations within the Tumors Exhibit the BCSC Phenotype and High Levels of Proliferative Markers

**Figure 5:**
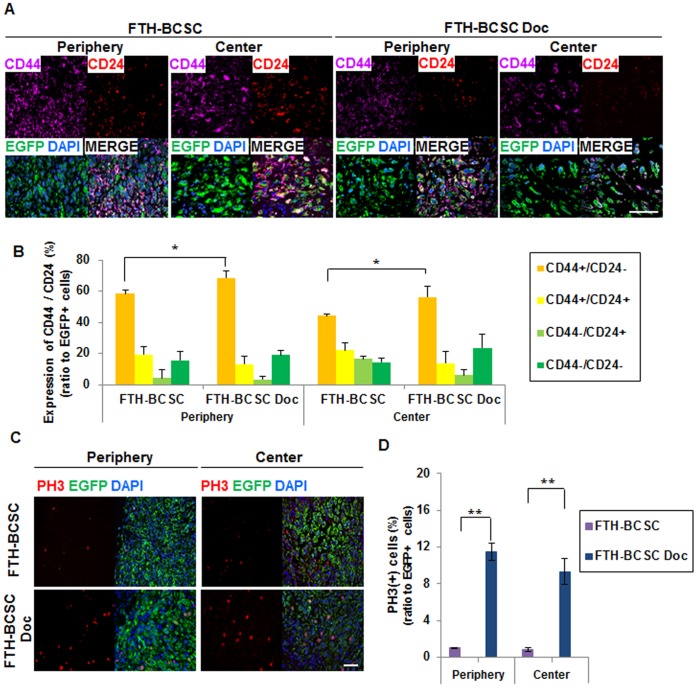
Immunohistochemical analysis of the peripheral and central portions of control (FTH-BCSC) and docetaxel-treated (FTH-BCSC Doc) xenograft tumors. (A) Double staining for CD44 and CD24 expression performed in FTH-BCSC and FTH-BCSC Doc tumors (x400; bar, 100 µm). (B) The percentage of CD44+/CD24−, CD44+/CD24+, CD44−/CD24+, CD44−/CD24− and EGFP-positive cells in the periphery and center of the FTH-BCSC and FTH-BCSC Doc tumors. *P<0.05. (C) Cell proliferation marker (phospho-histone 3, PH3) expression in the FTH-BCSC and FTH-BCSC Doc tumors (x200; bar, 100 µm). (D) The percentage of PH3-positive/EGFP-positive cells in the periphery and center of the FTH-BCSC and FTH-BCSC Doc tumors. **P<0.01.

To investigate the effects of docetaxel treatment on FTH-BCSC tumors, double staining for CD44 and CD24 was performed on FTH-BCSC and FTH-BCSC Doc tumors. Because the cells located in the periphery and center of the tumor responded differently to docetaxel, CD44 and CD24 expression was analyzed in both regions (Figure 5A). The periphery of the FTH-BCSC Doc tumors contained significantly higher proportions of CD44+/CD24− BCSCs compared to the periphery and center of the FTH-BCSC tumors (FTH-BCSC periphery and center: 58.46±2.07% and 44.35±4.82%; FTH-BCSC Doc periphery and center: 71.38±3.75% and 56.13±5.92%, *P*<0.05) (Figure 5B). CD44+/CD24+ and CD44−/CD24− cells were detected in the periphery and center of FTH-BCSC and FTH-BCSC Doc tumors. However, the proportion of these cells was not significantly different between FTH-BCSC and FTH-BCSC DOC tumors.

We investigated the cell proliferation marker PH3 to determine whether docetaxel alters tumor cell proliferation. The percentage of cells expressing PH3 in the EGFP-positive cell population was analyzed in the periphery and center of FTH-BCSC and FTH-BCSC Doc tumors. Significantly higher percentage of PH3-positive cells were observed in the periphery and center of the FTH-BCSC Doc tumors (FTH-BCSC periphery and center: 0.97±0.04% and 0.83±0.19%, FTH-BCSC Doc periphery and center: 11.48±0.90% and 9.32±1.38%, *P*<0.01) (Figure 5C and D).

## Discussion

The results of our study suggest that ferritin-based MRI can be used as a noninvasive method to identify viable cell populations in tumors after chemotherapy. In the present study, BCSCs transduced with FTH and EGFP dual reporter genes were transplanted into NOD/SCID mice to noninvasively track BCSC-derived populations during tumor growth and monitor tumor responses after chemotherapy. MRI showed distinct populations of pixels with high R_2_* values in the docetaxel-treated FTH-BCSC tumors, which correspond to EGFP+ viable cell populations with a high percentage of CD44+/CD24− cells, as observed by histology. We confirmed that lentiviral transduction of the reporter genes did not alter the characteristics of BSCSs, as was revealed by the cell surface marker analysis, proliferation assays, and *in vivo* tumor growth. To the best of our knowledge, this study is the first to track and image CSCs isolated from human tumor specimens and to show viable cell populations of tumors after chemotherapy in living mice with MRI reporter genes. We believe that our experimental model system can be used to identify the most effective treatments for tumors derived from BCSCs and to develop new therapeutic strategies to target both BCSCs and non-BCSCs to achieve durable remission [8], [9], [18], [32].

In general, accumulation of iron in the cancer cells may have adverse effects on the host since iron is pivotal nutrient for proliferation and growth in normal cells as well as cancer cells. Some studies reported high iron concentration promoted the cancer cell proliferation by overexpressing transferrin receptor that could elevate the level of reactive soluble iron in cells [33], [34]. However, the study reported by Cohen et al. [22] and our study demonstrated iron accumulation by ferritin overexpression did not alter breast cancer cell proliferation and viability *in vitro* [25]. These results suggest that ferritin overexpression can detoxify the reactive free iron not to affect the proliferation and growth of cancer cell, even the increase in net intracellular iron amount by inducing iron uptake.

A notable finding in this MRI study was the distinctive R_2_* value distribution found in FTH-BCSC tumors after chemotherapy. After 14 days of docetaxel treatment, a population of pixels with high R_2_* values appeared in the FTH-BCSC tumors while the mean R_2_* values of tumors were significantly decreased. In contrast, untreated BCSC, FTH-BCSC and docetaxel-treated BCSC tumors did not show this distinctive R_2_* value distribution. All R_2_* values for the control tumors showed a normal distribution. Pixels that represented a distinct population and measured over the threshold value (mean +3SDs) of the docetaxel-treated FTH-BCSC tumors occupied approximately 50% of the total pixels, while the percentages for control tumors were under 30%. These findings support the result that cell populations with different R_2_* value distributions were distinguished by FTH overexpression. Finally, tissue analysis confirmed that the viable cells in the FTH-BCSC tumors after chemotherapy were only located in regions with high R_2_* values, while the viable cells in the BCSC tumors were in both regions with low and high R_2_* values. Together, these findings imply that MRI analysis using a ferritin reporter can identify and quantify the existence of cells that survive after chemotherapy even before the tumor changes in size.

To monitor the BCSC tumor response to chemotherapy, our bimodal imaging approach based on FTH-EGFP dual reporter genes has advantages over optical imaging or MRI alone because areas of viable cells in tumors can be localized with 3D volumetric analysis of MRI data while the use of EGFP as a reporter gene enables the *in vitro* identification and molecular analysis of these viable cells [25], [27], [35], [36]. Tissue samples from tumors could be obtained without sacrifice of animals by using an MRI-guided biopsy system [37]. Furthermore, quantitative analyses of the MRI data enabled the evaluation of anti-tumor effects in different areas within the tumor, which is difficult with other *in vivo* methods. In this study, a higher percentage of cells with the CD44+/CD24− phenotype was found in the remaining viable cells in the docetaxel-treated FTH-BCSC tumors than in the untreated FTH-BCSC tumors. This result demonstrates that more cells with the BCSC phenotype are present in docetaxel-treated tumors, which is consistent with results of previous studies [6]. In the future clinical trials, the efficacy of CSC-targeting therapies is likely be monitored *in vivo* with MRI and SPIO nanoparticle-labeling of anti-CSC markers while our imaging approach with bimodal reporter genes is more suitable for studying dynamic processes or tumor cells to stroma interaction in preclinical animal tumor models [32], [38]–[41].

In conclusion, our results show that ferritin-based MRI, which offers high spatial resolution and tissue contrast, can effectively identify and localize remaining viable cell populations derived from BCSCs after chemotherapy and may represent a novel tool to monitor the efficacy of CSC-targeting therapies *in vivo*. The experimental model system used in this study could be easily applied to other cancer types, such as prostate, colon, pancreas, liver and brain tumors.

## Supporting Information

Figure S1 **Biological properties of the human BCSCs and FTH-BCSCs.** (A) The abilities to form mammospheres did not differ between BCSCs and FTH-BCSCs. (B) A BCSC marker (CD49f^high^), mesenchymal lineage markers (CD90^high^), and a hematopoietic stem cell marker (CD34−) were analyzed by flow cytometry in BCSCs. (C) CD marker expression levels of FTH-BCSCs were similar to those of BCSCs (CD49f^high^, CD90^high^ and CD34−).
(TIF)

Figure S2 **Comparison of tumor-forming abilities and fluorescence imaging of BCSCs and FTH-BCSC-derived tumors.** (A) Tumor-forming abilities were similar for BCSCs and FTH-BCSCs, and *in vivo* live imaging confirmed that only the FTH-BCSC tumors expressed EGFP fluorescence. The red dotted circle indicates the BCSC tumor and the yellow dotted circle indicates the FTH-BCSC tumor. (B) *Ex vivo* EGFP fluorescence of image excised tumors derived from BCSCs and FTH-BCSCs. The sizes of the BCSCs and FTH-BCSC tumors were similar.
(TIF)

Figure S3 **Cell growth analysis and the 7-AAD assay with iron supplementation.** (A) There was no significant difference in the growth rates of the BCSCs and FTH-BCSCs in the presence of an iron supplement (FAC). (B) The 7-AAD assay revealed that the viabilities of the BCSCs and FTH-BCSCs in the presence of the iron supplement were not significantly different.
(TIF)

Figure S4 **Growth rates of BCSC, FTH-BCSC, BCSC Doc and FTH-BCSC Doc tumors.** BCSCs and FTH-BCSCs (1×10^6^) were engrafted into the mammary fat pads of NOD/SCID mice. Mice (n = 5 per group) were treated with i.v. injections of docetaxel (15 mg/kg) at three-day intervals beginning the day after the pre-treatment MRI. Tumor growth was attenuated in BCSC Doc and FTH-BCSC Doc tumors 5 days after docetaxel treatment, and FTH overexpression did not affect the tumor growth rate in either the docetaxel-untreated or treated groups (BCSC vs. FTH-BCSC tumors, BCSC Doc vs. FTH-BCSC Doc tumors). The bars in the graph represent SDs.
(TIF)

Figure S5 Slice-by-slice analysis of R_2_* values in the xenograft tumors. Four representative slices from each group of tumors (BCSC, BCSC Doc, FTH-BCSC and FTH-BCSC Doc tumors) at day 0, day 5 and day 14 were processed with MATLAB. MRI Images in red box at day 14 were used in Figure 4. Color map range of R_2_* values: 0–160 (sec^−1^).
(TIF)

Figure S6 **Immunohistochemistry analysis of FTH in FTH-BCSC and FTH-BCSC Doc tumors.** H&E staining images and EGFP fluorescence images were analyzed in FTH-BCSC and FTH-BCSC Doc tumors. Viable portions of H&E staining and EGFP expressing cells that constituted the FTH-BCSC tumors and the viable portion of the FTH-BCSC Doc tumors were well matched. Magnification: H&E, 40×; and fluorescence, 40×. Scale bars: 200 µm.
(TIF)

Figure S7 ***In vitro*** **cytotoxicity test in BCSCs and FTH-BCSCs with docetaxel treatment.** (A) The percentages of JC-1 aggregates and cell viabilities after the docetaxel treatments were evaluated by the calculation of JC1 aggregates numbers on the fluorescence microscope images. (B) MTT assay was performed to evaluate the toxicity of docetaxel in BCSCs and FTH-BCSCs.
(TIF)

Table S1 R_2_* threshold values of docetaxel-untreated (BCSC and FTH-BCSC) and docetaxel-treated (BCSC Doc and FTH-BCSC Doc) xenograft tumors at day 0, day 5 and day 14.
(DOCX)

Protocol S1 Determination of R_2_* Threshold Values.
(DOCX)
