# Supplementary figures and images for: Noninvasive Identification of Viable Cell Populations in Docetaxel-Treated Breast Tumors Using Ferritin-Based Magnetic Resonance Imaging
Source: PLoS One. 2013 Jan 2;8(1):e52931. doi: 10.1371/journal.pone.0052931 (PMC3534651; doi:10.1371/journal.pone.0052931)

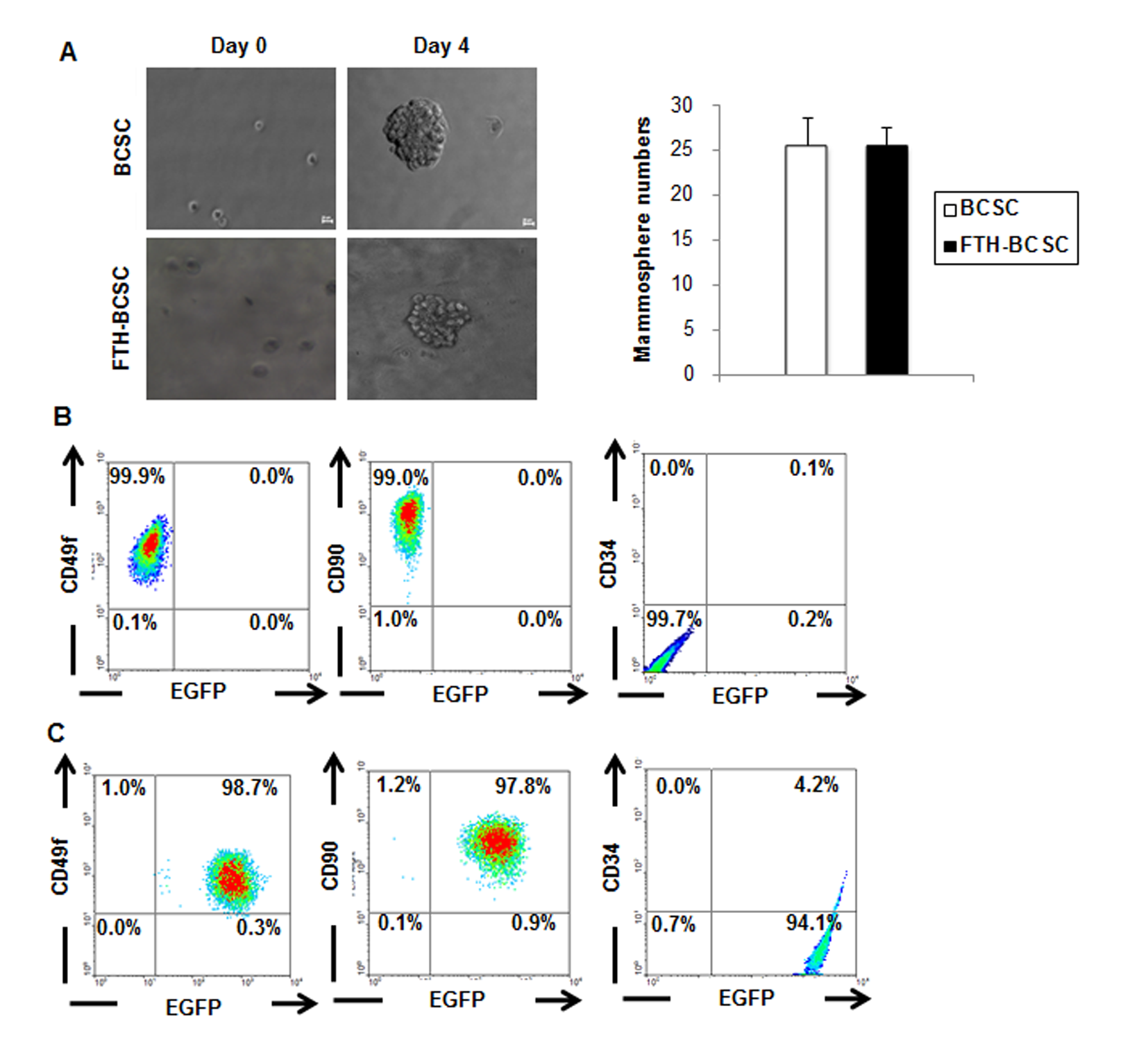

Supplement: Figure S1 — Biological properties of the human BCSCs and FTH-BCSCs. (A) The abilities to form mammospheres did not differ between BCSCs and FTH-BCSCs. (B) A BCSC marker (CD49fhigh), mesenchymal lineage markers (CD90high), and a hematopoietic stem cell marker (CD34−) were analyzed by flow cytometry in BCSCs. (C) CD marker expression levels of FTH-BCSCs were similar to those of BCSCs (CD49fhigh, CD90high and CD34−). (TIF) [file pone.0052931.s001.tif]

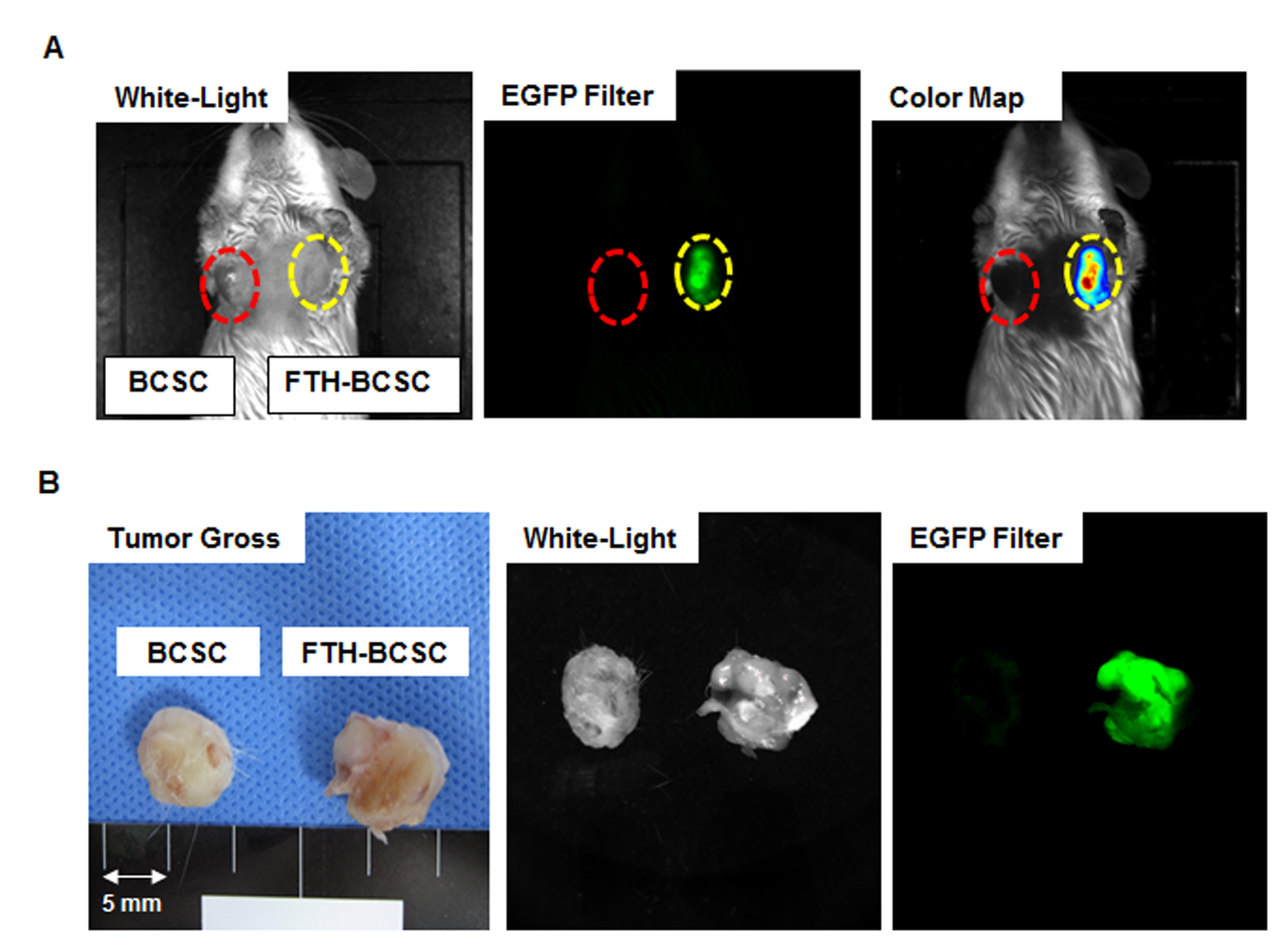

Supplement: Figure S2 — Comparison of tumor-forming abilities and fluorescence imaging of BCSCs and FTH-BCSC-derived tumors. (A) Tumor-forming abilities were similar for BCSCs and FTH-BCSCs, and in vivo live imaging confirmed that only the FTH-BCSC tumors expressed EGFP fluorescence. The red dotted circle indicates the BCSC tumor and the yellow dotted circle indicates the FTH-BCSC tumor. (B) Ex vivo EGFP fluorescence of image excised tumors derived from BCSCs and FTH-BCSCs. The sizes of the BCSCs and FTH-BCSC tumors were similar. (TIF) [file pone.0052931.s002.tif]

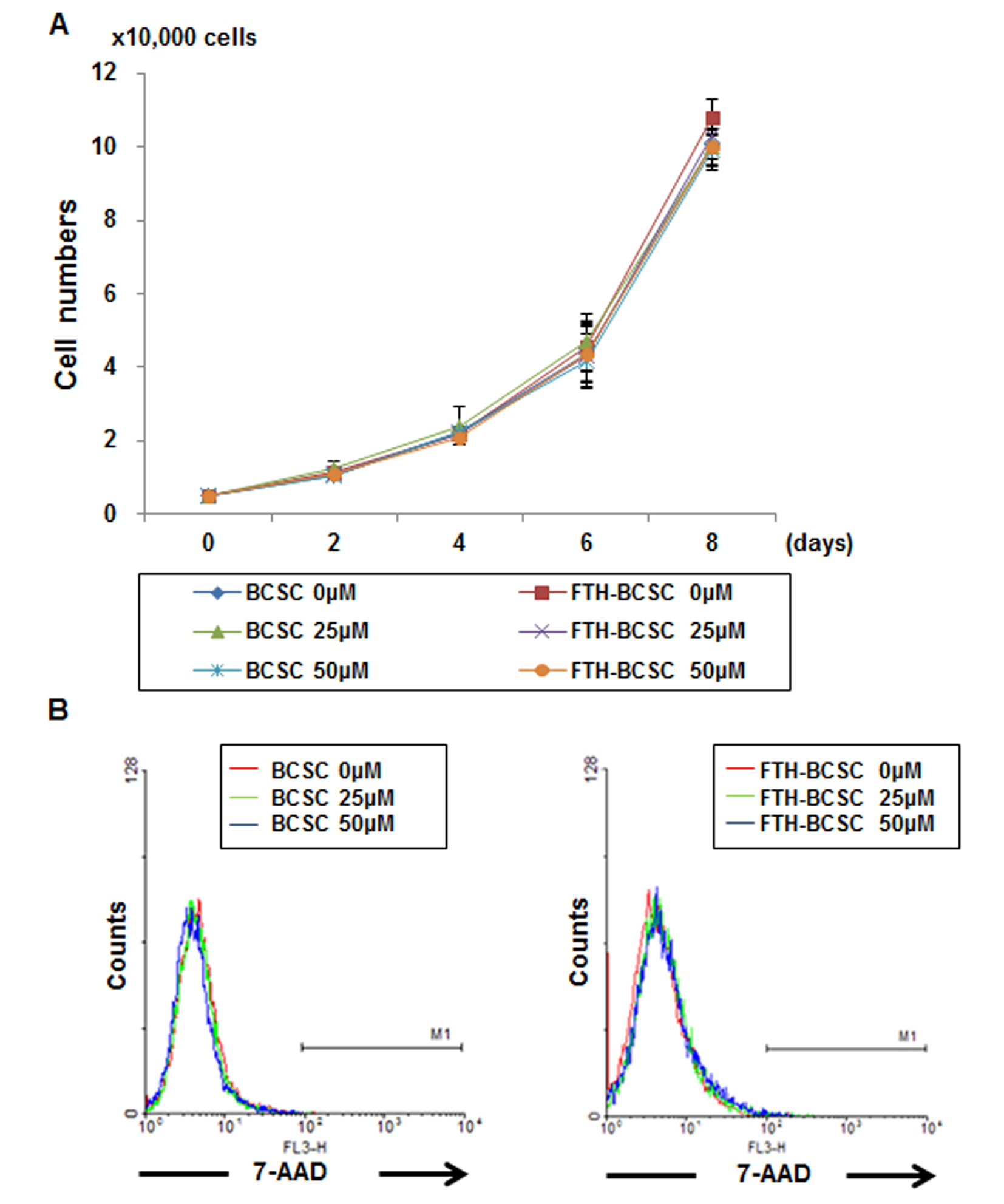

Supplement: Figure S3 — Cell growth analysis and the 7-AAD assay with iron supplementation. (A) There was no significant difference in the growth rates of the BCSCs and FTH-BCSCs in the presence of an iron supplement (FAC). (B) The 7-AAD assay revealed that the viabilities of the BCSCs and FTH-BCSCs in the presence of the iron supplement were not significantly different. (TIF) [file pone.0052931.s003.tif]

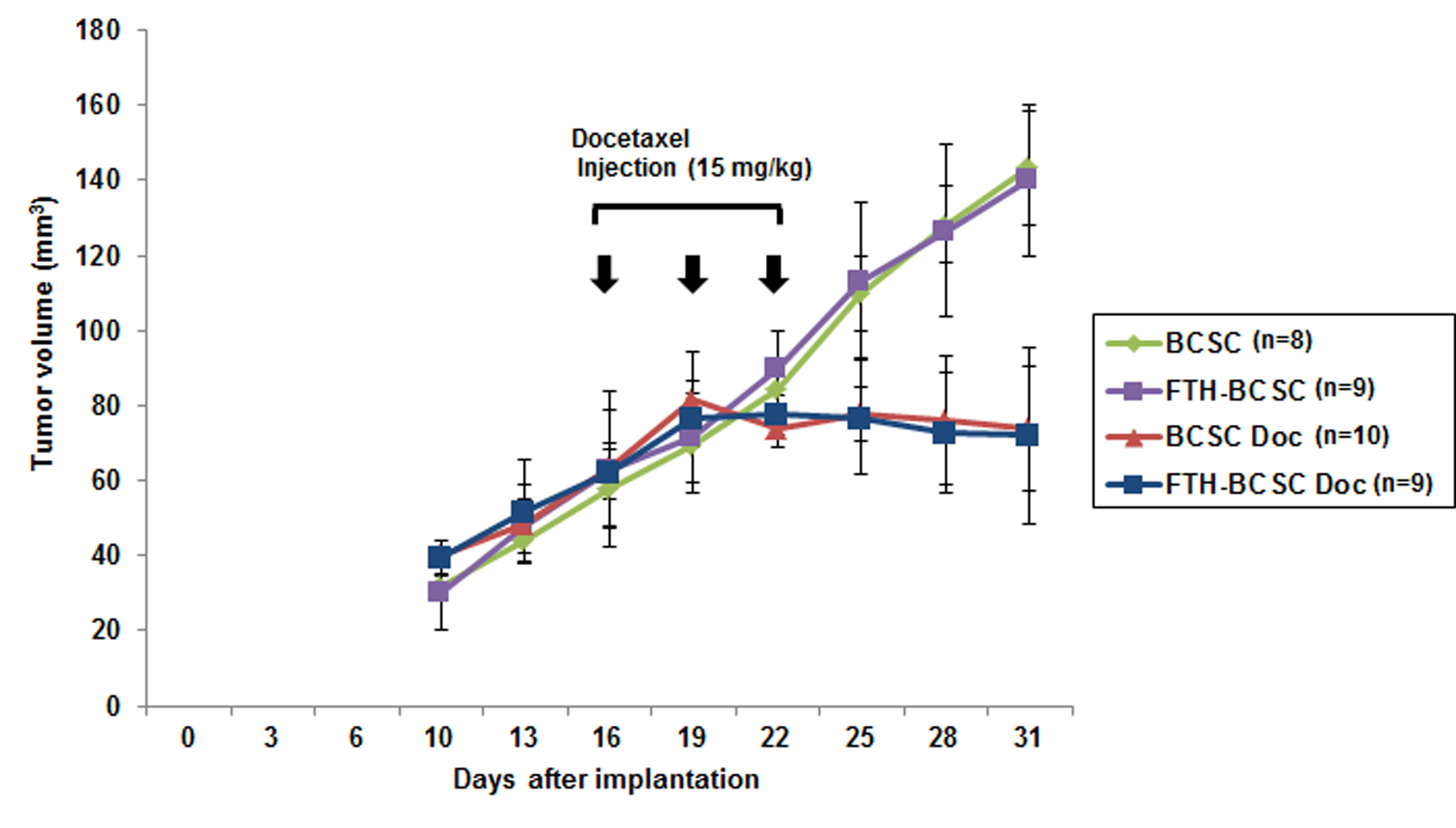

Supplement: Figure S4 — Growth rates of BCSC, FTH-BCSC, BCSC Doc and FTH-BCSC Doc tumors. BCSCs and FTH-BCSCs (1×10^6^) were engrafted into the mammary fat pads of NOD/SCID mice. Mice (n = 5 per group) were treated with i.v. injections of docetaxel (15 mg/kg) at three-day intervals beginning the day after the pre-treatment MRI. Tumor growth was attenuated in BCSC Doc and FTH-BCSC Doc tumors 5 days after docetaxel treatment, and FTH overexpression did not affect the tumor growth rate in either the docetaxel-untreated or treated groups (BCSC vs. FTH-BCSC tumors, BCSC Doc vs. FTH-BCSC Doc tumors). The bars in the graph represent SDs. (TIF) [file pone.0052931.s004.tif]

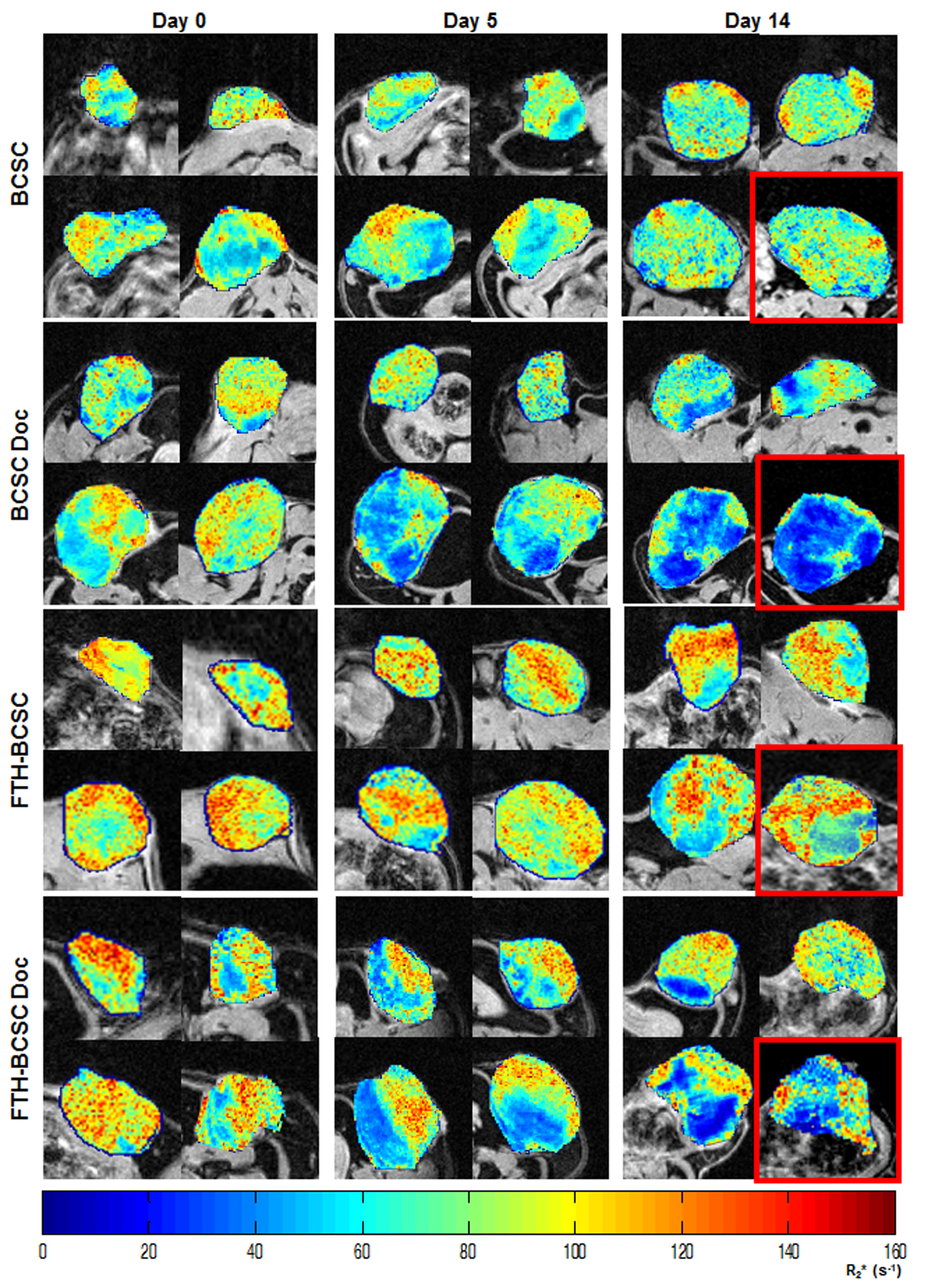

Supplement: Figure S5 — Slice-by-slice analysis of R2* values in the xenograft tumors. Four representative slices from each group of tumors (BCSC, BCSC Doc, FTH-BCSC and FTH-BCSC Doc tumors) at day 0, day 5 and day 14 were processed with MATLAB. MRI Images in red box at day 14 were used in Figure 4. Color map range of R2* values: 0–160 (sec−1). (TIF) [file pone.0052931.s005.tif]

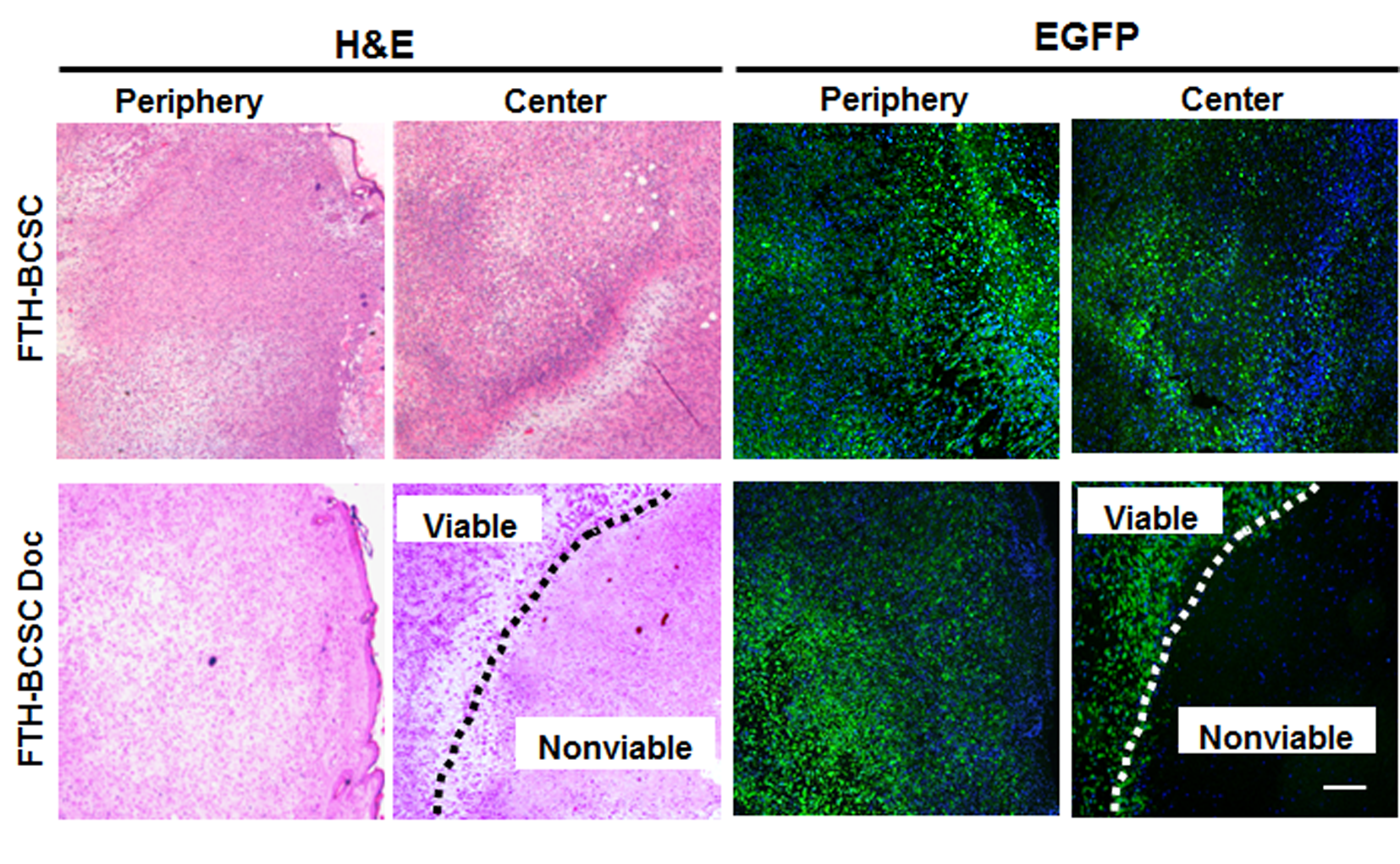

Supplement: Figure S6 — Immunohistochemistry analysis of FTH in FTH-BCSC and FTH-BCSC Doc tumors. H&E staining images and EGFP fluorescence images were analyzed in FTH-BCSC and FTH-BCSC Doc tumors. Viable portions of H&E staining and EGFP expressing cells that constituted the FTH-BCSC tumors and the viable portion of the FTH-BCSC Doc tumors were well matched. Magnification: H&E, 40×; and fluorescence, 40×. Scale bars: 200 µm. (TIF) [file pone.0052931.s006.tif]

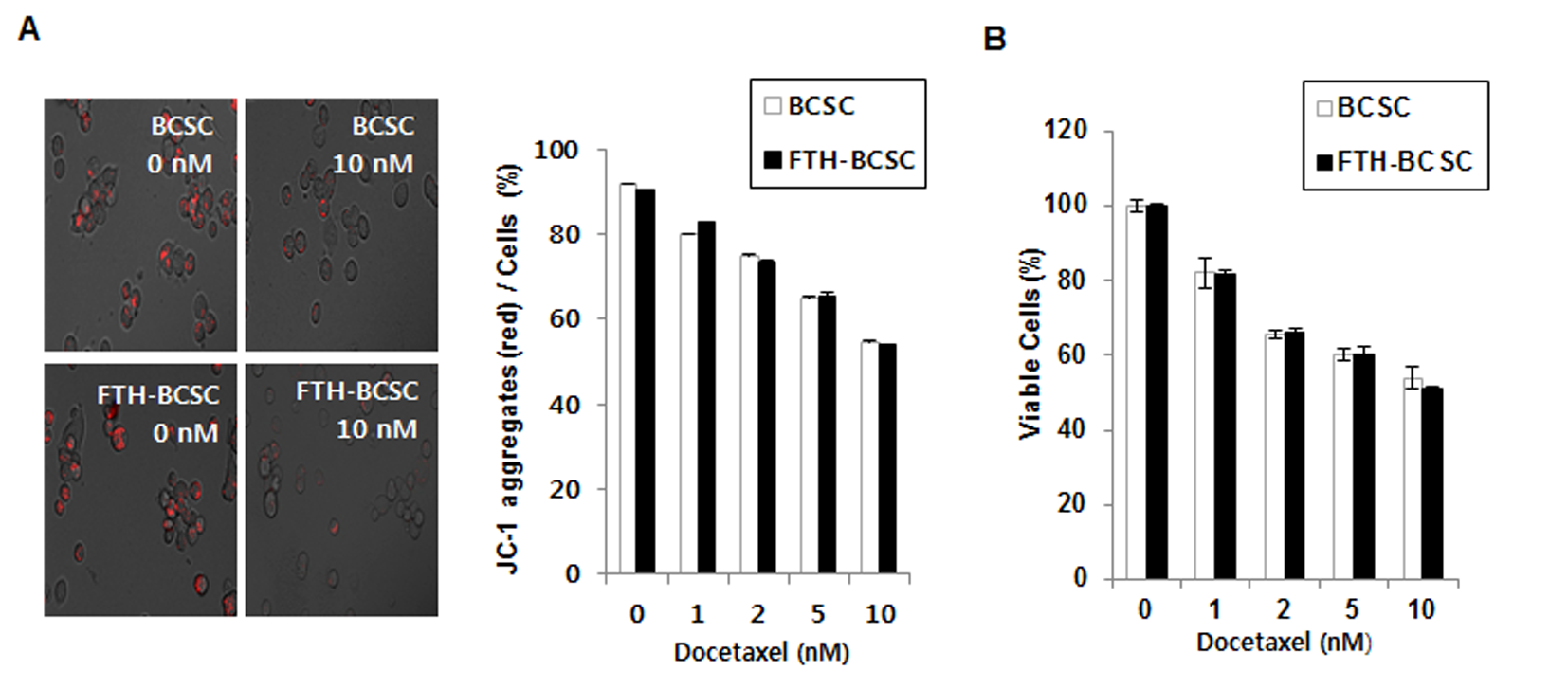

Supplement: Figure S7 — In vitro cytotoxicity test in BCSCs and FTH-BCSCs with docetaxel treatment. (A) The percentages of JC-1 aggregates and cell viabilities after the docetaxel treatments were evaluated by the calculation of JC1 aggregates numbers on the fluorescence microscope images. (B) MTT assay was performed to evaluate the toxicity of docetaxel in BCSCs and FTH-BCSCs. (TIF) [file pone.0052931.s007.tif]
