# Supplementary material for: Noninvasive Identification of Viable Cell Populations in Docetaxel-Treated Breast Tumors Using Ferritin-Based Magnetic Resonance Imaging
Source: PLoS One. 2013 Jan 2;8(1):e52931. doi: 10.1371/journal.pone.0052931 (PMC3534651; doi:10.1371/journal.pone.0052931)
Supplement: Table S1 — R2* threshold values of docetaxel-untreated (BCSC and FTH-BCSC) and docetaxel-treated (BCSC Doc and FTH-BCSC Doc) xenograft tumors at day 0, day 5 and day 14. (DOCX) [file pone.0052931.s008.docx]

**Supporting Table 1. R_2_* threshold values of docetaxel-untreated (BCSC and FTH-BCSC) and docetaxel-treated (BCSC Doc and FTH-BCSC Doc) xenograft tumors at day 0, day 5 and day 14**

| Tumors | Day 0 | Day 5 | Day 14 |
| --- | --- | --- | --- |
| BCSC | 102.92 | 103.99 | 104.38 |
| FTH-BCSC | 122.3 | 119.59 | 114.4 |
| BCSC Doc | 104.38 | 77.97 | 67.01 |
| FTH-BCSC Doc | 122.54 | 95.15 | 82.75 |
