# Supplementary material for: Noninvasive Identification of Viable Cell Populations in Docetaxel-Treated Breast Tumors Using Ferritin-Based Magnetic Resonance Imaging
Source: PLoS One. 2013 Jan 2;8(1):e52931. doi: 10.1371/journal.pone.0052931 (PMC3534651; doi:10.1371/journal.pone.0052931)
Supplement: Protocol S1 — Determination of R2* Threshold Values. (DOCX) [file pone.0052931.s009.docx]

**Supporting Protocol S1**

The R_2_* distribution of the entire histogram was obtained by calculating the values from all tumor slices of each group with intervals of three slices. Then, the pixel percentage of the R_2_* values greater than the mean + SD (mean plus standard deviation), mean + 2SDs and mean + 3SDs values of each group was obtained by dividing the number of pixels with the values corresponding to mean + SD, mean + 2SDs and mean + 3SDs in the tumor histograms of each group by the number of pixels in all of the histograms and subsequently multiplying that number by 100. The mean + SD, mean + 2SDs and mean + 3SDs pixel percentages of each group were calculated (data not shown), and the mean + 3SDs value that best represented the skewedness of the FTH-BCSC Doc tumors was defined as the threshold.
